# The Rules of Engagement: Perspectives on Secure Messaging From Experienced Ambulatory Patient Portal Users

**DOI:** 10.2196/medinform.7516

**Published:** 2017-07-04

**Authors:** Cynthia J Sieck, Jennifer L Hefner, Jeanette Schnierle, Hannah Florian, Aradhna Agarwal, Kristen Rundell, Ann Scheck McAlearney

**Affiliations:** ^1^ The Ohio State University College of Medicine Columbus, OH United States; ^2^ The Ohio State University College of Public Health Division of Health Services Management and Policy Columbus, OH United States

**Keywords:** patient portals, secure messaging, patient-provider communication, qualitative study

## Abstract

**Background:**

Patient portals have shown promise in engaging individuals in self-management of chronic conditions by allowing patients to input and track health information and exchange secure electronic messages with their providers. Past studies have identified patient barriers to portal use including usability issues, low health literacy, and concerns about loss of personal contact as well as provider concerns such as increased time spent responding to messages. However, to date, studies of both patient and provider perspectives on portal use have focused on the pre-implementation or initial implementation phases and do not consider how these issues may change as patients and providers gain greater experience with portals.

**Objective:**

Our study examined the following research question: Within primary care offices with high rates of patient-portal use, what do experienced physician and patient users of the ambulatory portal perceive as the benefits and challenges of portal use in general and secure messaging in particular?

**Methods:**

This qualitative study involved 42 interviews with experienced physician and patient users of an ambulatory patient portal, Epic’s MyChart. Participants were recruited from the Department of Family Medicine at a large Academic Medical Center (AMC) and included providers and their patients, who had been diagnosed with at least one chronic condition. A total of 29 patients and 13 primary care physicians participated in the interviews. All interviews were conducted by telephone and followed a semistructured interview guide. Interviews were transcribed verbatim to permit rigorous qualitative analysis. Both inductive and deductive methods were used to code and analyze the data iteratively, paying particular attention to themes involving secure messaging.

**Results:**

Experienced portal users discussed several emergent themes related to a need for greater clarity on when and how to use the secure messaging feature. Patient concerns included worry about imposing on their physician’s time, the lack of provider compensation for responding to secure messages, and uncertainty about when to use secure messaging to communicate with their providers. Similarly, providers articulated a lack of clarity as to the appropriate way to communicate via MyChart and suggested that additional training for both patients and providers might be important. Patient training could include orienting patients to the “rules of engagement” at portal sign-up, either in the office or through an online tutorial.

**Conclusions:**

As secure messaging through patient portals is increasingly being used as a method of physician-patient communication, both patients and providers are looking for guidance on how to appropriately engage with each other using this tool. Patients worry about whether their use is appropriate, and providers are concerned about the content of messages, which allow them to effectively manage patient questions. Our findings suggest that additional training may help address the concerns of both patients and providers, by providing “rules of engagement” for communication via patient portals.

## Introduction

Patient portals provide access to information in the patient’s electronic health record, in addition to serving as a platform to view and schedule appointments and engage in secure communication with providers [1]. These types of portals, typically accessed by patients through a website, are increasingly positioned as a central component of patient engagement in healthcare [2-5]. Specifically, portals have shown promise in engaging individuals in self-management of chronic conditions by allowing patients to input and track health information, facilitating communication between patients and providers, and providing access to consumer-friendly information about diseases [6-10].

One particular feature of patient portals, secure messaging, has seen a significant increase in use over time [11,12]. This popular feature allows patients and providers to communicate asynchronously, without waiting for the other to be available on the telephone. For example, through secure messaging, patients can provide updates on symptoms discussed during a visit or efficiently monitor the initiation of some types of medications. The literature suggests that secure messaging can facilitate access to care, improve patient satisfaction, and improve health outcomes [13,14]. Unlike the other features of a patient portal, such as viewing lab and test results or requesting appointments, secure messaging allows for the exchange of direct communication between patients and providers. As a result, studies suggest that a patient’s relationship with a provider is a key predictor of the patient’s intention to use secure messaging [15].

While secure messaging is a function generally desired by patients, both patients and providers share concerns about its use. Some worry about the loss of interpersonal contact [16-18] as well as about the privacy and security of information exchanged through a patient portal [17,19-22]. Additionally, providers have expressed concerns about the impact of secure messaging on their workload [20,23-26], noting that they are typically not reimbursed for this type of work [26-29].

One important limitation of this literature on patient portals is that studies of patient and provider perspectives on portal use focus on the pre-implementation or initial implementation phases and do not reflect how use and perspectives may change as users gain more experience. For example, surveys or interviews of providers are typically conducted before the implementation of the portal to gauge providers’ willingness to accept the portal and inform decisions about portal design [30-33] or immediately after portal implementation [34-38]. A 2016 study of primary care providers’ views on patient portals published in the *Journal of Medical Internet Research* included only 7 current portal users among the 20 interviewees [23]. The same is true for patients, with most qualitative studies involving only early or recent patient-portal adopters rather than experienced users [6,21,39,40]; as a result, these studies are frequently focused on barriers to adoption [17,41-46].

While the perspectives of patients and providers are critical in the early phases of patient-portal implementation and use, there is a gap in the literature regarding how experienced users, both patients and providers, engage with portals and use secure messaging features. Our study aimed to address this gap by exploring the following research question: Within primary care offices with high rates of patient-portal use, what do experienced physician and patient users of the ambulatory portal perceive as the benefits and challenges of portal use in general and secure messaging in particular? Interviewing both physicians and patients with use experience allowed us to consider questions such as whether privacy and security are still prominent patient concerns among active, long-term users, and whether provider’s work flow concerns persist once portal use is established within the office.

## Methods

### Study Design

We designed an exploratory qualitative study to improve our understanding of patients’ and providers’ perspectives on patient portals and the use of secure messaging within those portals. Our data were collected through telephone interviews with participants recruited for the study. Data were then iteratively analyzed, using both deductive and inductive methods, to characterize the themes we present in this paper. This study was approved by the study site’s Institutional Review Board.

### Study Setting

Our study took place at a large Midwestern Academic Medical Center (AMC) that uses Epic’s MyChart, an interactive tethered patient portal that allows patients to view test and lab results, schedule appointments, request refills, and send secure messages to providers. Patients using the portal are presented with a notice on the secure messaging screen (1) telling them to use this feature for non-urgent messages only, (2) telling them to expect a response within 24-48 hours, (3) reminding them that their message becomes part of their medical record, and (4) telling them to call 911 if they feel their concern represents an emergency.

Since implementing MyChart across the entire AMC in 2012, over 35,000 patients have created a MyChart account, with the majority having logged on at least once. The demographics of portal users are skewed toward greater representation by females, whites, and patients between the ages of 36 and 54. Of the MyChart features available, messaging and viewing results are the most commonly used, followed by appointment scheduling. Across all departments in the AMC, Family Medicine providers have the highest percentage of active MyChart users (65% of their patients), followed by Obstetrics/Gynecology (55%); other departments average between 35%-50%.

### Study Sample

We recruited a purposive sample of patients and primary care physicians in the summer and fall of 2015. Interviewees were all experienced users of MyChart and included 13 Family Medicine providers in the Department of Family Medicine (DFM) and 29 of their patients who had at least one chronic condition. Patients were identified by their physician using the reporting function of the electronic health record (EHR). Inclusion criteria were having at least one cardiopulmonary condition and being among the most frequent users of MyChart when patients were rank ordered by frequency of message. Providers forwarded a recruitment e-mail from the study principal investigator (PI) to the top 25 frequent users identified in their query. The recruitment email explained the purpose of the study and provided a contact number for patients to call to schedule telephone interviews. Providers were recruited to participate in interviews through a similar e-mail sent directly from the study PI. Interviews lasted approximately 30 minutes, and all interviews were conducted by telephone and recorded.

### Data Collection

We used two versions of a semistructured interview guide to conduct the interviews, drawing upon concerns about using portals identified in our literature review [16-29], as well as our own research questions related to the portal user experience. Interview questions for patients asked about motivations for using MyChart, how patients use MyChart, and perceptions about how MyChart impacts patient-provider communication. Providers were asked about the primary activities they completed on MyChart and their experiences with these activities, including releasing lab results and fielding patient questions via the portal. Providers were also asked about perceived impacts on the patient-provider relationship and challenges to engaging with patients through MyChart. Interviews were transcribed verbatim to permit rigorous analysis.

### Analysis

Our analytic approach used both inductive and deductive methods iteratively, using a constant comparative analytic approach throughout the study [47]. First, a three-person coding team identified broad themes from the data and developed a preliminary non-mutually exclusive coding dictionary. This team also proposed new codes as patterns emerged from the data and as subsequent interviews were conducted, following the methods described by Constas [48]. While the three-person team made initial coding decisions, frequent meetings with the entire study team were held to discuss discrepancies, reach consensus, and ensure that saturation of concepts was reached. We used the Atlas.ti (version 6.0) qualitative data analysis software to support our analysis.

## Results

We conducted 42 interviews of 29 patients and 13 primary care physicians. Our qualitative analysis of interview transcripts revealed five major themes related to the use of secure messaging within the patient portal, as well as a theme involving providers’ perspectives about the need for training on portal use. Below we describe these themes related to benefits and concerns about secure messaging, including sub-themes about concerns from the perspectives of patients and providers, respectively. We conclude with an exploration of sub-themes around the need for “rules of engagement” to support portal use.

### Perceived Benefits of Secure Messaging

#### Asynchronous Communication

Both patients and providers appreciated the ability to use secure messaging for communication. Most commonly, both groups felt that the ability for each party to respond according to their own schedule increased the efficiency of communicating. Several patients specifically mentioned the benefit of conversations that could occur asynchronously, according to the patient’s and provider’s individual schedules, without reliance on telephone calls to the office. For example, one patient described communication via MyChart in comparison with how he had to call the office before using MyChart:

If I had a question for them, I would call in and deal with what seems to be a number of [people]. First you talk to the receptionist, and then you get to the nurse, and then you try to do the medication option. And call back when you get lost in the line of communication there some way.

Providers also described this benefit and noted increased efficiency in communications. A provider described it thus:

Because sometimes, when it’s a phone call, I’m not necessarily making the call. I let my staff do it. So it goes from me, to the staff, to the patient. So this way [using messaging in MyChart] I get straight to the patient. So it’s a lot quicker.

#### Electronic Record of Communication

In addition to facilitating communication, patients also discussed the benefits of having an electronic record of exchanges with their provider. A patient told us this:

It’s just I can go in and access the message. I have a written copy, too, of what was said which, again to me getting older, is enormously important for me to have something I can go back to and go, ‘Now, what did he say about that?’

Another patient described having this electronic record in a similar manner, as MyChart was perceived to help focus the office visit:

I think it helps us more to focus on things. I can come in and say, ‘Oh hey doc, I saw your note.’ So when I am in the office, we already kind of got an idea of what is going on most of the time. And when I am out of the office, through MyChart, I can actually keep up on things. I just feel like the doctor knows better what is going on with me, and is able to respond to my situation quicker.

#### Provider Preferences in the Use of Secure Messaging

At the same time, we noted variations in the expectations and attitudes of providers toward using secure messaging and in the ways they address this topic with patients. For instance, when asked how they determined whether a patient needed an office visit or not, some providers offered clear guidance while others were more equivocal. A provider described how having the portal was a helpful way to remotely manage a patient’s chronic conditions via secure messages:

I will say, ‘I want you to check your blood pressure, once a week for the next month, email me the results and then we can decide what we need to do from there.’ Whereas before, I would have had to have them come back and show me their results on paper.

However, another noted that this benefit would depend on a variety of factors:

Oh geez...it depends probably on the complexity of the problem. There are some problems that I would say, even though it’s a new problem, a problem that I’ve never seen, sending a MyChart message to me seems totally appropriate.

While providers reportedly appreciated this possibility, they noted that more information was needed about portal use. As one summarized it:

I think, that it would be good to have a little more education.

### Perceived Concerns About Secure Messaging

#### Patients’ Concerns About Secure Messaging

Three subthemes emerged involving patients’ concerns about the secure messaging feature in the patient portal: (1) concern about imposing on the physician, (2) concern about lack of compensation for the provider, and (3) confusion about when to use the feature. Each of these sub-themes is further explained below, with additional evidence supporting these findings presented in Table 1.

##### Imposing on Providers’ Time

Some patients were concerned that they would be taking up too much of their provider’s time if they sent messages via the portal instead of going to the office to meet in person. A patient explained it as follows:

I try to make sure that I only use it for important things. Or things that I know they want to know about. Well, like when I contact the doctor about getting labs before I come in, that is a useful thing. But, I am not going to contact one of my specialists in the middle, or 6 months away from an appointment just to say, hey I have this little itch or something.

Patients were also reportedly uncertain about how much messaging was too much, noting that they did not want to be a nuisance or a bother. A patient remarked:

...my biggest fear is that I don’t want to get to a point where I am annoying the doctor and sending him three messages every day or something.

Another patient had similar thoughts:

But I try not to interrupt. She’s got a life...and this is a new thing for me. I don’t want to be a nuisance.

##### Uncompensated Provider Time

Patients also reported concern about the fact that messaging a provider via the portal could result in uncompensated time for the provider. For example, one patient stated:

So yeah, there have been times when I might have gone up for an appointment and I got enough answers through MyChart that I did not. So yeah, in one sense that’s good for me that it prevented a trip, you know. For the business of medicine, I don’t know.

Another patient similarly acknowledged the lack of provider reimbursement for interactions on MyChart:

...otherwise I would’ve had to go in and this is a business after all.

##### Lack of Clarity About When to Send a Secure Message

Patients in our study also noted that they were often uncertain about when it is appropriate to use the messaging feature to communicate with their physician. While most recognized that emergency situations were inappropriate, there was considerable lack of clarity as to what to do in non-emergent situations. As one patient described their thoughts:

If everything is stable, I could probably go three months without using it. It’s more when something is stirred up, which is, as I get older, that happens more frequently. And, you know, it’s just a transitional time of life when, ‘I don’t even know if that’s normal or not. Should I come in for that or am I wasting your time?’

Another patient echoed this sentiment, noting:

That is the hard part.

#### Providers’ Concerns About Secure Messaging

Three subthemes also emerged involving providers’ concerns about the secure messaging feature: (1) concern about unfocused or insufficient information in the messages, (2) concern about inappropriate message topics, and (3) concern about incorrect use of the secure messaging feature. Here, we describe these sub-themes in greater detail, with additional supporting evidence provided in Table 1.

##### Unfocused or Insufficient Information in Messages

Most frequently, providers noted that patient messages did not contain sufficient information upon which they could make a recommendation, despite the messages sometimes being quite lengthy. A provider gave us an example of this lack of clarity:

I may get 10 to 15 messages constantly in 2-3 hours from the same patient. ‘Okay...I am feeling fatigued for 2 weeks.’ So you know, that is not enough information for me. So I ask, ‘Okay, do you have any other symptoms or do you want to see me?’ And in the end you are lost, because you need to see the patient.

**Table 1:** Patient and provider concerns about secure messaging.

Concerns		Representative verbatim comment
**Patient concerns**		
	Imposing on provider’s time	“Try to keep it to the important stuff and if I need to be seen, then make an appointment, at least that is what I am trying to do.”
		“I mean, I try to use...leave my physicians alone because, you know, I know that they have, you know, their number one priority is to take care of patients that are in the office.”
		“I didn’t want to be a pain in the arse to all the doctors by, you know, trying to ask them so many questions.”
	Uncompensated provider time	“And you know, sometimes I think, well I feel bad that I don’t go in and give him his due for his time. But you know, this only took a second or two.”
		“It was just that he would take the time to read it and respond without like coming in and paying for an appointment just increased my trust, I guess, that when a lot of things these days seem to be for the money, he had my well-being in mind.”
	Uncertainty about when to use the portal	“Yeah and it’s like I say it's at his convenience for that. So he’s not rushed, and I’m not taking away from anything.”
		“Yeah, I don’t know if I should be using it for that purpose, I don’t know how much of his time I should take up.”
**Provider concerns**		
	Unfocused and/or insufficient information	“I mean I have had people, I can think of one in particular. A guy sent in about a 4-paragraph message, detailing numerous complaints, I’m not sure what he expected, but my answer was like, ‘This is much too complicated, you have to come to the office.’”
		“So, to get valid information from patients, over the Internet probably requires a little bit more education than a lot of our patients have. Because if you can’t accurately describe symptoms, then you can’t accurately describe what you are doing, then it is going to be really hard to manage this appropriately. It is really hard to manage things appropriate regardless, but over MyChart, the degree of difficulty just increases.”
		“Well that again, some of my people they’ll go on and on. I have another colleague whose patient will go on and on even more than mine. And when it gets to a point you probably need to have a conversation back and forth, you probably need a face to face conversation, I try to set up an appointment.”
	Inappropriate topic	“Yeah one of the big pitfalls of MyChart messaging is the chest pain message. So, I have had people message, ‘I have been having left side chest pain radiating to my arm, I get short of breath, what should I do?’ So, these messages, we are not sitting by the computer waiting for the message to come in. I saw her message 4 hours later, I just happened to be going on, because I was on-call on a Saturday. And then I had to call first thing, didn’t answer, so it created a big crisis really. But it ended up that she was okay. And I had to get her son to go to her house, and he ended up taking her to the ER, and everything turned out fine. But at the time we didn’t know.”
		“They want to give you this litany follow up, of what has been happening at home, you know, like you are email buddies. I don’t like being any patient’s email buddy.”
	Incorrect use of message feature	“I guess I don’t like when it is used incorrectly, emergencies, for clerical issues, things that should be handled by another staff member that doesn’t need to go directly to me. More and more the message comes to me and no one deals with it or answers the question. The patient just feels empowered to say, ‘Hey I need to schedule an appointment,’ It took me like two minutes to open it up figure it out and send a message, close it give it to someone else.”
		“‘Can you check on my prescription for something,’ and normally a nurse would be able to do that without me even knowing about it or getting involved. But now I have to get involved. I have to do it all.”
		“The patients can make appointments but, they often don’t click on the right button so those come to us.”

Another provider reflected, *“they will write paragraphs.”* Even with long messages, however, providers were concerned about the quality of the information provided. As one provider noted, long descriptions without a clear question were of concern:

...writing pages and paragraphs, to give you the history of their problem. The history should come in a visit, not a question. That is not a question.

##### Inappropriate Message Topic

Providers were also concerned that patients would send them messages via MyChart that were inappropriate for that mode. For instance, one provider explained how a patient would add detail that was not about the patient himself or herself:

I don’t like it when patients, like a family member will send, for example a mother will say, ‘Johnny got a fever today,’ and she sends it on her chart. And that happens a lot. And it sort of contaminates her chart. And now we have information, confidential information, cause it can get like ‘Well, you know my husband, you know his diabetes is worse now, and blah blah blah,’ and now it is on the wife’s chart. So, now Johnny Smith’s diabetes information is on Susie Smith’s chart. And for me that is like a confidentiality breach.

Similarly, several providers we interviewed felt patients treated messages as informal, friendly communications. A provider explained this with an example:

...like my patients, they send me a picture from India. Like ‘Hi, we are having fun from India, just wanted to say hello…’ This is not a public email. It’s nice to chat, but that is not the purpose of MyChart.

##### Incorrect Use of Message Feature

Another area of concern raised by providers was incorrect use of the MyChart secure messaging feature. For instance, several providers complained that patients would use the secure messaging feature directly to request an appointment, rather than the “schedule an appointment” button. A provider explained this:

A patient says, ‘I want to see you for an appointment. Please schedule me,’ and stuff like that. I don’t do scheduling.

Another incorrect use of the secure messaging feature emerged in the context of requesting refills. As one provider explained:

...people send refills on MyChart, and I don’t mean the refill mechanism, but they message me with a refill.

#### Providers’ Suggestions to Improve Patient-Portal Use

From providers’ suggestions on how to improve use of the secure messaging feature in the patient portal, an important theme emerged. Taken together, these comments suggested an important opportunity to clarify the “rules of engagement” for a patient portal. We identified three sub-themes in this area, related to how patient-portal use could be improved by providing guidance on these “rules” as well as how the feature could be enhanced to reinforce the “rules”: (1) offer patient training on appropriate portal use, (2) make patients accountable for learning how to use the portal, and (3) enhance the secure messaging feature to reinforce the “rules.” We describe these sub-themes below and present additional supportive quotes in Table 2.

##### Offer Patient Training on Appropriate Portal Use

To address provider concerns about how patients use the portal, some providers suggested developing instructions or training for patients focused on how to use MyChart appropriately to communicate efficiently and effectively with their providers. Providers noted that this training would need to address issues beyond the technical aspects of how to navigate within MyChart and suggested the opportunity to emphasize the “rules of engagement” with a patient portal. For instance, this content would need to provide directions on how to communicate via the portal, including when to use secure messages versus when to call or schedule an appointment. A provider summarized it:

So, to get valid information from patients over the Internet probably requires a little bit more education than a lot of our patients have. Because if you can’t accurately describe symptoms, then you can’t accurately describe what you are doing, then it is going to be really hard to manage this appropriately.

Similarly, another provider suggested:

...to make sure the communication is more effective and more productive is something that probably could be trained.

**Table 2:** Opportunities to clarify “rules of engagement” and improve patient-portal use.

Providers’ suggestions	Representative verbatim comment
Offer patient training on appropriate patient-portal use	“One thing that I think might be helpful is to have like almost guidelines for the patient, of what kinds of things are appropriate for MyChart and what kind of things aren’t. So, you know, this is not to discuss new problems or symptoms you are having. That needs to be an office visit. It is to follow up, for quick questions. That kind of thing.”
	“...with training patients and probably providers to some extent too, on how to use it appropriately and transmit the appropriate information.”
Make patients accountable for learning how to use the patient portal	“When they sign up, if we have it written on paper or something like that, that we can hand them and say, ‘Please review these guidelines.’ Maybe have them initial off that they have read them.”
	“Electronically, like have a course. They can take a course, like very brief course. And sign an agreement. And after they sign the agreement, and they understand the application of MyChart, then they would be allowed to sign in for MyChart.”
Enhance secure messaging feature to reinforce “rules”	“I think that when people send refills on MyChart, and I don’t mean the refill mechanism, but they message me with a refill. So it might be good if there was a pop up saying, ‘There is another way to do refills,’ ‘There is another way for emergencies, which is to call on-call,’ ‘There is another way to if it is not about you go to that person’s MyChart.’ So it might be good to have some kind of pop-up, just so they stop and read. It could probably save a lot of nonsense messages.”
	“I think it would be great if it could be filtered, through some system or people. Or some messages need to go to the desk, scheduling person, somewhere. It should go directly to them rather than coming to me and I have to answer and route it to them.”

##### Make Patients Accountable for Learning How to Use the Patient Portal

Providers also noted the importance of making patients responsible for learning how to properly use the portal. These providers suggested that there might be different opportunities to provide the training, including at portal sign up or during a visit, but emphasized that patients should be held accountable for this learning. More than one provider suggested the drafting of a document that patients would be asked to sign, acknowledging receipt of this education, and noted they would then be able to refer to the document later when discussing appropriate messaging during future visits. A provider proposed this:

The patient can read the agreement, and you know click on it. And then, you know, you can go to the patient and they can sign up for MyChart. And we have a document saying, listen you have read this and you cannot use it like that.

##### Enhance Secure Messaging Feature

Providers proposed several opportunities to enhance the MyChart application functionality in ways that could automatically provide guidance to patients within the secure messaging feature. Of these, one opportunity was around providing information about the urgency of the message. A provider suggested the following solution:

I think that when they open it up to send a message, it should say like hang on a minute, are you complaining about an emergency situation? It is like when they call our office and the message says if it is an emergency, call 9-1-1. And maybe there needs to be something, a pop-up, saying, ‘Are you sure that this is the appropriate medium?’

Another enhancement proposed was that secure messages could be limited to a certain number of characters. A provider told us this:

The university has a policy that, for any message, you need to limit it to so many characters. And when they get too much characters, the university says—sends them a little note saying, ‘Sorry, but with the use of this, we need to limit the amount of information in this due to your physician’s need to address all his patient’s concerns.’

Providers similarly commented about opportunities to provide direction to patients about the appropriateness of message content around refills and appointment scheduling, suggesting that pop-up messages or other portal enhancements might work.

## Discussion

### Overall Findings

Our study suggests that initial concerns about overuse and security of information expressed by patients and providers in pre-implementation studies [19,23-26] may no longer apply as users gain experience. Instead, experienced users identified concerns beyond the technical aspects of using a portal. Patients worried about imposing on a provider’s time, uncompensated provider time, and a lack of clarity about when to send a secure message. Providers did not discuss an increased workload as has been noted in pre-implementation studies [20,23-26]; instead, they were concerned about unfocused and/or insufficient information in messages, inappropriate message topics, and incorrect use of the message feature. In discussing these concerns, providers suggested a need for further training focused on these issues.

The portal used in this setting provides patients with instructions, described in the Study Setting above, about when to use a secure message to set patient expectations about response times and provides some guidance on whether to send a message or call 911. However, patients we interviewed expressed confusion about how to define non-urgent concerns, and providers noted that some patients still included information in their messages that was inappropriate for their medical record.

Unlike other portal features such as scheduling appointments or requesting prescription refills, secure messaging requires interaction with another individual and therefore users need to understand more than simply the technical aspects of how to access a feature. Appropriate use requires an understanding of the type of information that should be conveyed via the portal and the etiquette rules of electronic communication. Yet, little guidance is provided to patients or providers related to the “rules of engagement” in secure messaging.

### Practice Recommendations

Our findings suggest that information and training on the “rules of engagement” is needed on several levels. For patients, print materials and instructional videos can be presented as they begin to use a portal. Such materials can provide patients with information about creating an account and navigating through the portal’s features. However, additional training and information related to how to engage and communicate via a portal may be required to improve communication for both patients and providers, particularly for experienced users such as those we interviewed.

Patient-focused information could be developed to set the tone for the “rules of engagement” and address issues such as when secure messaging is appropriate, question topics that can be addressed via secure messaging, what type of information to include in the messages, and how to understand information sent by the provider. Additionally, such material represents another opportunity to address patient safety by reminding patients that their provider may not see the message immediately, and, therefore, secure messaging should not be used for emergency situations. This information would thus provide patients with guidance on how to engage with, and not just how to navigate, the portal, thereby potentially alleviating patient concerns related to perceived burden as well as facilitating more efficient communication within the portal.

Providers could also benefit from clarifying the “rules of engagement” from their perspective. Currently, providers may receive training on the aspects of the patient portal that face them as providers, such as how to view and send a secure message. Additional training that exposes providers to the patient view of the portal may provide a more complete understanding of the patient experience and help them to better interact with their patients. In addition, guidance could be provided on how to communicate in secure messages or alongside lab and test results. Past studies of patient-provider communication have focused mainly on in-person communication, with electronic communication studied primarily to document trends in use [11-14]. Therefore, providers, like patients, typically have little guidance on the language they could use in portal communication or how to structure such communications. In addition, unlike in a face-to-face encounter, electronic communications make it difficult for providers to assess patient comprehension. Training providers to send better messages may increase the quality of patient-provider communication and reduce the need for additional clarifying messages. Topics this training could address include communicating positive and negative results, communicating at the appropriate level of health literacy, and providing educational materials to facilitate patient understanding.

At the same time, providers also need to establish clear and consistent guidelines of the expectations they have for patients in communicating via a patient portal. Before the patient portal was implemented, patients would call their provider’s office with questions. While this process had its own inefficiencies, such as waiting time on the telephone or leaving and returning phone messages, information was most often filtered through office staff who had general knowledge about the information a physician would need to respond to that particular question. Communication via a patient portal, however, lacks such a filter to focus patient questions and the information they convey. In addition, secure messaging is asynchronous and therefore may lack the conversational nature of an in-person visit in which information can be exchanged and clarified quickly. Further, our study demonstrates that even patients experienced in patient portal use lack clarity on when to use a secure message and what information to include. Similarly, while some providers in our study mentioned preparing patients to receive lab or test results via the patient portal, none discussed communication expectations with patients. In our study, we note that these expectations may vary by individual provider, suggesting that discussions about portal use may help to improve the efficiency of patient-provider communication and alleviate patient concerns about being a burden to their providers.

In practice, portal technology could leverage electronic communication capabilities by incorporating features such as built-in guidance. For example, as physician interviewees suggested, including a link on the secure messaging screen to guide patients in determining whether their concern meets the criteria for being “non-urgent” could be helpful. Furthermore, developing structured message boxes to guide patients to complete the information providers need to address patient concerns may not only help ensure that necessary information is conveyed, but also help patients focus their messages and more clearly describe their concerns.

As patients, providers, and health care systems gain greater experience with patient portals, new needs emerge to define the “rules of engagement” through a portal. While there are a range of technical solutions that could be implemented to improve patient and provider communication via secure messaging, it is important to elicit input from all stakeholders in designing these modifications. The patients in our study, who were experienced users, had clear thoughts on what they liked about the secure messaging and identified specific areas in which they were uncertain about how to use this tool. Discussions with patients can help to further refine their concerns and develop new ways to address them. As noted above, for the most part, providers in our study did not express the concerns noted in the literature in pre-implementation studies, specifically related to the increased workload of secure messaging. However, they identified areas in which the process of secure messaging could be improved. Further work is needed to develop stakeholder-driven solutions to these issues. While our study did not include healthcare system administrators, they play a significant role in encouraging the use of patient portals in general and secure messaging in particular. Their goals for secure messaging could also be important in shaping the next round of education and training to clarify the “rules of engagement.”

### Limitations

We note the inclusion of only one health system as a limitation of our study. Although the features of the patient portal used by this health system are common to those used across the country, the experiences of interviewees in our study are limited to how the portal has been implemented and used in this health system. While we reached saturation on the topics covered in our interviews, patients and providers in other health systems or using other patient portals may have different perspectives. Additionally, as is typical in qualitative studies, we did not collect demographic data from the interviewees. Differing perspectives by demographic characteristics may be explored in future studies.

### Conclusions

As patients and providers gain more experience with patient portals, the needs and perspectives of both groups regarding portals are evolving. Many patients are now beyond the “new user” phase and are realizing the benefits of more comprehensive portal use. Communication through portals is increasingly viewed as an extension of care between visits. While we can expect that this will result in better management of patient conditions, our study demonstrates new concerns that arise with greater use. Patients struggle to balance their desire to respect their provider’s time with their need for answers to health-related questions. Providers are still figuring out how best to communicate with patients via portals in a way that addresses patient needs without overstepping boundaries. These findings suggest that additional information and training on the “rules of engagement” may help address the concerns of both patients and providers and improve the efficiency of communication via patient portals.

## Abbreviations

AMC: Academic Medical Center
HER: electronic health record
PI: principal investigator
